# The assessment of replicability using the sum of *p*-values

**DOI:** 10.1098/rsos.240149

**Published:** 2024-08-28

**Authors:** Leonhard Held, Samuel Pawel, Charlotte Micheloud

**Affiliations:** ^1^ Epidemiology Biostatistics and Prevention Institute (EBPI) and Center for Reproducible Science (CRS), University of Zurich, Hirschengraben 84, Zurich 8001, Switzerland

**Keywords:** Edgington’s method, *p*-values, replication studies, sample size planning, two-trials rule, Type-I error rate

## Abstract

Statistical significance of both the original and the replication study is a commonly used criterion to assess replication attempts, also known as the two-trials rule in drug development. However, replication studies are sometimes conducted although the original study is non-significant, in which case Type-I error rate control across both studies is no longer guaranteed. We propose an alternative method to assess replicability using the sum of p -values from the two studies. The approach provides a combined p -value and can be calibrated to control the overall Type-I error rate at the same level as the two-trials rule but allows for replication success even if the original study is non-significant. The unweighted version requires a less restrictive level of significance at replication if the original study is already convincing which facilitates sample size reductions of up to 10%. Downweighting the original study accounts for possible bias and requires a more stringent significance level and larger sample sizes at replication. Data from four large-scale replication projects are used to illustrate and compare the proposed method with the two-trials rule, meta-analysis and Fisher’s combination method.

## 1. Introduction

Replication studies are increasingly conducted in various fields to assess the replicability of original findings. While ‘replicability’ is intuitively understood as the ability to obtain consistent results when a study is repeated with new subjects [1], there is no agreed-upon quantitative definition of replicability or ‘replication success’. In practice, a variety of different approaches are used that capture different intuitions about what it means for a replication to be successful. For example, metrics such as the Q -test or prediction intervals quantify the statistical compatibility of the effect estimates from original and replication studies [2,3] while a meta-analysis of original and replication results provides an assessment of the evidence pooled across both studies [4].

Perhaps the most commonly used success criterion is the statistical significance of the replication study with an effect estimate in the same direction as in the original study [5–9]. Positive original findings are usually also based on significance, and the procedure is then analogous to the ‘two-trials rule’ in drug development [10, §12.2.8]. The intuition behind this approach is that each study individually should provide sufficient evidence for a claim, which is not guaranteed with other approaches such as meta-analysis or the Q -test. In practice, the standard two-sided significance level 0.05 is often used and so replication success with the two-trials rule is achieved if both one-sided p -values, $p_{o}$ and $p_{r}$ , from the original and replication study, respectively, are smaller than α=0.05/2=0.025 . The one-sided formulation is convenient to ensure that the effect estimates from the two studies are in the same direction.

The success condition of the two-trials rule $\max\{p_{o},p_{r}\}\leq \alpha$ can be rewritten as


(1.1) $$p_{2TR}=\max\{p_{o},p_{r}{\}}^{2}\leq \alpha^{2}\;,$$

where the combined p -value $p_{2TR}$ of the two-trials rule turns out to be valid [11], i.e. uniformly distributed under the intersection null hypothesis that both the true effect $\theta_{o}$ in the original study and the true effect $\theta_{r}$ in the replication study are null. The two-trials rule therefore controls the *overall Type-I error rate* at level $\alpha^{2}$ . For α=0.025 , the probability to incorrectly declare replication success under the intersection null hypothesis is $0.025^{2}=0.000625$ .

Despite its simplicity, in the replication setting the two-trials rule has important limitations. First of all, the ‘double dichotomization’ at α=0.025 can lead to conclusions which seem counterintuitive. For example, the two-trials rule is not fulfilled if $p_{o}=0.026$ and $p_{r}=0.001$ (or vice versa), but it is fulfilled when both $p_{o}$ and $p_{r}$ are 0.024, although there is less evidence for an effect in the second case [12]. The first example also illustrates that replication success is impossible if the original study just missed statistical significance, no matter how convincing the replication study is. However, some replication projects do interpret non-significant original studies as positive findings and try to replicate them. In the *Reproducibility Project: Psychology* (*RPP*) [5], four ’positive’, but non-significant original effects have been included, all with (one-sided) p -values between 0.025 and 0.03. In the *Experimental Economics Replication Project* (*EERP*) [6], two ’positive’, but non-significant effects have been included, one with $p_{o}=0.027$ [13] and one with an even larger p -value, $p_{o}=0.035$ [14]. The strict application of the two-trials rule would inflate the overall Type-I error rate and reduce trust in the replication finding.

The past and ongoing debate on the use and misuse of p -values [15–17] has led various researchers to advocate for a quantitative interpretation of p -values as measures of the strength of evidence [18] or divergence [19]. This implies that an original study with a relatively large p -value carries only suggestive evidence against the null hypothesis of no effect [20] and requires a more convincing replication result for confirmation than an original study with a smaller p -value. There is in fact empirical evidence that studies with small p -values tend to replicate better than studies with only suggestive evidence. For example, several large-scale replication projects found strong negative Spearman correlations between original p -values and the corresponding (two-sided) replication p -values being smaller than 0.05 [5–7]. Held [21] has shown that more stringent significance levels have improved performance in predicting replication success in an application to the data from the Open Science Collaboration [5] project. The two-trials rule, however, requires the same level of evidence at replication no matter what level of evidence the claim of the original discovery had, as long as it was significant.

A promising alternative is to summarize the total evidence from the two p -values with a combined p -value, but different from the one based on the two-trials rule (1.1). A large number of valid p -value combination methods are available [22,23] and the two-trials rule can serve as a benchmark as it provides the success threshold $\alpha^{2}$ to ensure appropriate control of the overall Type-I error rate [24–26]. Perhaps most prominent is Fisher’s combination method [27], where replication success at level $\alpha^{2}$ is achieved if the product of the p -values $p_{o}p_{r}\leq c_{F}=\exp\{-0.5\chi_{4}^{2}(1-\alpha^{2})\}$ , where $\chi_{\nu }^{2}(\cdot )$ denotes the quantile function of the $\chi^{2}$ -distribution with ν degrees of freedom. The corresponding combined p -value is


(1.2) $$p_{F}=1-\mathrm{Pr}\left(X\leq -2\log\{p_{o}p_{r}\}\right),$$

where X follows a $\chi^{2}$ distribution with 4 d.f., so replication success would be declared if $p_{F}\leq \alpha^{2}$ . However, the goal of replication studies is to confirm an original (positive) finding, but replication success with Fisher’s method can be achieved even if one of the p -values is very large. In fact, replication success at level $\alpha^{2}=0.025^{2}$ is guaranteed if $p_{o}<c_{F}\approx 0.00006$ , no matter how large $p_{r}$ is, so Fisher’s method may not even require a replication study to confirm a claim of a new discovery, which is clearly undesirable.

An alternative approach used often in replication projects [5–7,9] is to calculate a combined p -value $p_{\text{MA}}$ with a fixed-effect meta-analysis, also known as the ‘pooled-trials rule’ in drug development [10, §12.2.8]. Let Φ(⋅) denote the standard normal cumulative distribution function and $\Phi^{-1}(\cdot )$ the corresponding quantile function. The meta-analytic p -value $p_{\text{MA}}=1-\Phi (z_{\text{MA}})$ is identical to the combined p -value from weighted Stouffer’s method [23,28], where


(1.3) $$z_{\text{MA}}=\frac{\sigma_{r}\;z_{o}+\sigma_{o}\;z_{r}}{\sqrt{\sigma_{o}^{2}+\sigma_{r}^{2}}}\;$$

is the weighted average of the original and replication z -values $z_{o}=\Phi^{-1}(1-p_{o})$ and $z_{r}=\Phi^{-1}(1-p_{r})$ with weights proportional to the standard errors $\sigma_{r}$ and $\sigma_{o}$ of ${\hat{\theta }}_{r}$ and ${\hat{\theta }}_{o}$ , respectively. Again, the criterion $p_{\text{MA}}\leq \alpha^{2}$ has been used to assess replication success [24,25,29,30], although a threshold larger than 0.000625 (e.g. two-sided 0.005 or even 0.05) is often used in applications. But it is immediate from (1.3) that meta-analysis has similar problems Fisher’s method because $z_{\text{MA}}$ can become large (and hence $p_{\text{MA}}$ small) even if one of the two underlying z -values $z_{o}$ and $z_{r}$ is small or even negative (and hence the corresponding p -value large). Recently, Muradchanian *et al.* [4] have conducted a study to investigate meta-analysis as a replication success metric. They also conclude that meta-analysis is an inappropriate tool if one wants to evaluate whether the replication result is in line with the original result.

In what follows we propose a particular p -value combination method based on the sum of p -values which requires both studies to be convincing, similar to the two-trials rule: Edgington’s method [31] recently rediscovered by Held [32] in the context of drug regulation. For overall Type-I error control at level $\alpha^{2}=0.025^{2}$ , success is achieved if the sum of p -values is smaller than $\sqrt{2}\times 0.025\approx 0.035$ , so possible if either $p_{o}$ or $p_{r}$ is not significant, as long as they are both smaller than 0.035 . As such, the method can be used in the scenarios encountered in previous replication projects where an original study was borderline non-significant. We also derive a weighted version of Edgington’s method that treats original and replication differently. Giving twice the weight to the replication study results in the necessary (but not sufficient) success conditions $p_{o}\leq 2\;\alpha$ and $p_{r}\leq \alpha$ . Both versions of Edgington’s method reach opposite conclusions than the two-trials rule for the two examples introduced earlier: replication success at level $0.025^{2}$ is declared if $p_{o}=0.026$ and $p_{r}=0.001$ , but not if $p_{o}$ and $p_{r}$ are 0.024. The approach is summarized in box 1.

Box 1.
**Assessing replicability using the sum of *p*-values**

Overall Type-I error control at level $\alpha^{2}=0.025^{2}$ .

Input: one-sided p -values $p_{o}$ and $p_{r}$ from original and replication study.

**Table:** 

	unweighted	weighted
weights	$w_{o}=1$ , $w_{r}=1$	$w_{o}=1$ , $w_{r}=2$
replication success criterion	$\begin{array}{rcl} p_{o}+p_{r} & \leq & \sqrt{2}\;\alpha \\ & \approx & 0.035 \end{array}$	$\begin{array}{rcl} p_{o}+2\;p_{r} & \leq & 2\;\alpha \\ & = & 0.05 \end{array}$
significance level for replication study	$\begin{array}{rl} & \sqrt{2}\;\alpha -p_{o}\\ \approx & 0.035-p_{o} \end{array}$	$\begin{array}{rl} & \alpha -p_{o}/2\\ = & 0.025-p_{o}/2 \end{array}$
combined p -value	$p_{E}=(p_{o}+p_{r}{)}^{2}/2$ (for $p_{o}+p_{r}\leq 1$ )	$p_{E_{w}}=(p_{o}+2\;p_{r}{)}^{2}/4$ (for $p_{o}+2\;p_{r}\leq 1$ )

Other choices can be made for α and the weights $w_{o}$ and $w_{r}$ .

The rest of the article is organized as follows. Edgington’s method is described in §2.1 and extended to include weights in §2.2. In §3, we discuss and compare the *conditional Type-I error rate* and the *project power* of the two-trials rule, meta-analysis and Fisher’s and Edgington’s methods. The conditional Type-I error rate is the probability, given the original study result, to incorrectly declare replication success when the true effect is null at replication, while the project power is the probability to correctly declare replication success over both studies in combination. The different methods are applied to the data from four large-scale replication projects in §4 and sample size planning of the replication study is described in §5. Extensions to more than one replication study are described in §6. Finally, some discussion is provided in §7.

## 2. Additive combination of *p*-values

### 2.1. Edgington’s method

Edgington [31] developed a method to combine p -values by adding them. Here, we investigate the use of Edgington’s method in the replication setting, where results from one original and one replication study are available. Under the intersection null hypothesis, the sum of the two p -values:


(2.1) $$E=p_{o}+p_{r}$$

follows the Irwin–Hall distribution with parameter n=2 [33,34], i.e. E∼ IH(2). The cumulative distribution function of the Irwin–Hall distribution can then be used to calculate a valid combined p -value:


(2.2) $$\begin{array}{l} p_{E}=\mathrm{Pr}(\text{IH}(2)\leq E)=\left\{ \begin{array}{rl} E^{2}/2 & \text{ if }0<E\leq 1\;,\\ -1+2\;E-E^{2}/2 & \text{ if }1<E\leq 2\;. \end{array}\right. \end{array}$$

The success condition $p_{E}\leq \alpha^{2}$ can be expressed in terms of the p -value sum E≤b , where the critical value is $b=\sqrt{2}\;\alpha$ . At the standard α=0.025 , the critical value is hence b≈0.035 . The threshold b can be considered as the available budget for the two p -values, $p_{o}$ and $p_{r}$ , and this implies that replication success is possible for a non-significant original (or replication) p -value as long as $p_{o}$ (or $p_{r}$ ) <b=0.035 . The sum of the p -values for the two examples presented in §1 is E=0.026+0.001=0.027 and E=0.024+0.024=0.048 , respectively, so replication success is declared in the first example but not the second, in contrast to the two-trials rule but in accordance with intuition. The corresponding combination p -values (2.2) are $p_{E}=0.0004$ and $p_{E}=0.001$ , respectively, the first smaller and the second larger than the threshold $\alpha^{2}=0.000625$ .

### 2.2. Weighted version

An interesting extension of Edgington’s method is to include weights, for example, proportional to the precision of the studies, as in weighted Stouffer’s method. However, in the replication setting one might want to downweight the original and upweight the replication study, for example, with weights 1/3 and 2/3 , respectively. This would address concerns that original studies may be subject to questionable research practice and hence prone to bias. Consider the weighted sum of p -values:


(2.3) $$E_{w}=w_{o}p_{o}+w_{r}p_{r},$$

with positive weights $w_{o}\leq w_{r}$ , then we show in appendix A that the corresponding combination p -value is


(2.4) $$\begin{array}{l} p_{E_{w}}=\left\{ \begin{array}{rl} \frac{E_{w}^{2}}{2\;w_{o}w_{r}} & \text{ if }0<E_{w}\leq w_{o}\;,\\ \frac{1}{w_{r}}\left(E_{w}-\frac{w_{o}}{2}\right) & \text{ if }w_{o}<E_{w}\leq w_{r}\;,\\ 1+\frac{1}{w_{o}w_{r}}\left(E_{w}(w_{o}+w_{r})-\frac{(w_{o}+w_{r}{)}^{2}}{2}-\frac{E_{w}^{2}}{2}\right) & \text{ if }w_{r}<E_{w}\leq w_{o}+w_{r}\;. \end{array}\right. \end{array}$$

Note that (2.4) reduces to (2.2) for $w_{o}=w_{r}=1$ , and is invariant under multiplication of the weights $\left(w_{o},w_{r}\right)$ with a positive constant. The p -value (2.4) therefore only depends on the weight ratio $\tilde{w}=w_{r}/w_{o}$ of the replication to the original weight. Setting the first line of (2.4) equal to $\alpha^{2}$ gives the available budget $b_{w}=\sqrt{2w_{o}w_{r}}\;\alpha$ for $E_{w}\leq w_{o}$ .

In the following we will use the weights $w_{o}=1$ and $w_{r}=2$ , although other choices can be made, of course. Then


(2.5) $$\begin{array}{l} p_{E_{w}}=\left\{ \begin{array}{rl} E_{w}^{2}/4 & \text{ if }0<E_{w}\leq 1\;,\\ E_{w}/2-1/4 & \text{ if }1<E_{w}\leq 2\;,\\ -\frac{5}{4}+\frac{3}{2}E_{w}-E_{w}^{2}/4 & \text{ if }2<E_{w}\leq 3\;. \end{array}\right. \end{array}$$

For $E_{w}\leq 1$ with small enough $p_{o}<p_{r}$ , we have $p_{E_{w}}=(p_{o}+2\;p_{r}{)}^{2}/4\approx p_{r}^{2}=p_{2TR}$ from (1.1), so weighted Edgington will behave similar to the two-trials rule, whereas the p -value from the unweighted version will then be roughly half as large as $p_{2TR}$ : $p_{E}=(p_{o}+p_{r}{)}^{2}/2\approx p_{r}^{2}/2=p_{2TR}/2$ .

The success condition $p_{E_{w}}=E_{w}^{2}/4\leq \alpha^{2}$ can be rewritten as $E_{w}=p_{o}+2\;p_{r}\leq 2\;\alpha$ , so doubling the replication weight increases the budget from $\sqrt{2}\;\alpha$ to 2α and for α=0.025 it will be possible to successfully replicate original studies with $p_{o}\leq 0.05$ . However, the p -value of the replication study now counts twice in $E_{w}$ , so replication success is impossible if $p_{r}>\alpha$ , just as with the two-trials rule. For example, the original study by Kuziemko *et al.* [14] mentioned in §1 had a quite large p -value: $p_{o}=0.035$ . Conducting a replication would be pointless if analysis is based on the two-trials rule or even unweighted Edgington. However, replication success would be still possible with weighted Edgington, but the replication study has to be quite convincing to achieve success ( $p_{r}<0.025-0.035/2=0.0075$ ).

## 3. Operating characteristics

As discussed before, all methods considered control the overall Type-I error rate at level $\alpha^{2}$ . We will now look at two other operating characteristics: conditional Type-I error rate and project power.

### 3.1. Conditional Type-I error rate

The success condition on the replication p -value $p_{r}$ with the two-trials rule is always $p_{r}\leq \alpha$ , regardless of the original study result (as long as $p_{o}\leq \alpha$ holds). In contrast, with Edgington’s and Fisher’s methods as well as the meta-analysis criterion the required value of $p_{r}$ depends on the original p -value $p_{o}$ . This condition is


(3.1) $$p_{r}\leq b-p_{o}\;$$

for Edgington’s method,


(3.2) $$p_{r}\leq (b_{w}-w_{o}p_{o})/w_{r}\;$$

for weighted Edgington’s method,


(3.3) $$p_{r}\leq \min\{c_{F}/p_{o},1\}\;$$

for Fisher’s method, and


(3.4) $$p_{r}\leq 1-\Phi \left\{\frac{\Phi^{-1}(1-\alpha^{2})\sqrt{c+1}-z_{o}}{\sqrt{c}}\right\}\;$$

for the meta-analysis criterion, which depends on the variance ratio $c=\sigma_{o}^{2}/\sigma_{r}^{2}$ . For a very convincing original study (where $p_{o}\to 0$ , equivalently $z_{o}\to \infty$ ), the right-hand side in (3.1) tends to $b=\sqrt{2}\;\alpha$ , in (3.2) to $b_{w}/w_{r}=\sqrt{2/\tilde{w}}\;\alpha$ , while the right-hand side in (3.3) and (3.4) converges to 1 . Replication success can thus be achieved with Fisher’s method and the meta-analysis criterion even if the replication p -value is very large, while this cannot happen with the two-trials rule and Edgington’s method.

Of particular interest in the replication setting is the conditional Type-I error rate, the probability, given the original study result, that the replication study flags success although there is no true effect at replication ( $\theta_{r}=0$ ). For α=0.025 , the conditional Type-I error rate is α=0.025 , $b-p_{o}<b=0.035$ and $(b_{w}-w_{o}p_{o})/w_{r}<b_{w}/w_{r}=0.025$ with the two-trials rule and the unweighted and weighted Edgington’s method, respectively, but can become very large with Fisher’s method and the meta-analysis criterion, if $p_{o}$ is small. For example, suppose the original study had a p -value of $p_{o}=0.001$ . The conditional Type-I error rate of Edgington’s method at the standard α=0.025 level then is 3.4% (unweighted) and 2.45% (weighted). With Fisher’s method and meta-analysis (for c=1 ), the conditional Type-I error rate is 5.8% and 7.0%, respectively. Now suppose the original study had a p -value of $p_{o}=0.0001$ . The conditional Type-I error rate then is 3.53% (unweighted) and 2.495% (weighted) with Edgington’s method, so only slightly larger. However, with Fisher’s method and meta-analysis, the conditional Type-I error rate increases drastically to 58.1% and 19.9%, respectively.

### 3.2. Project power

**Figure 1.**
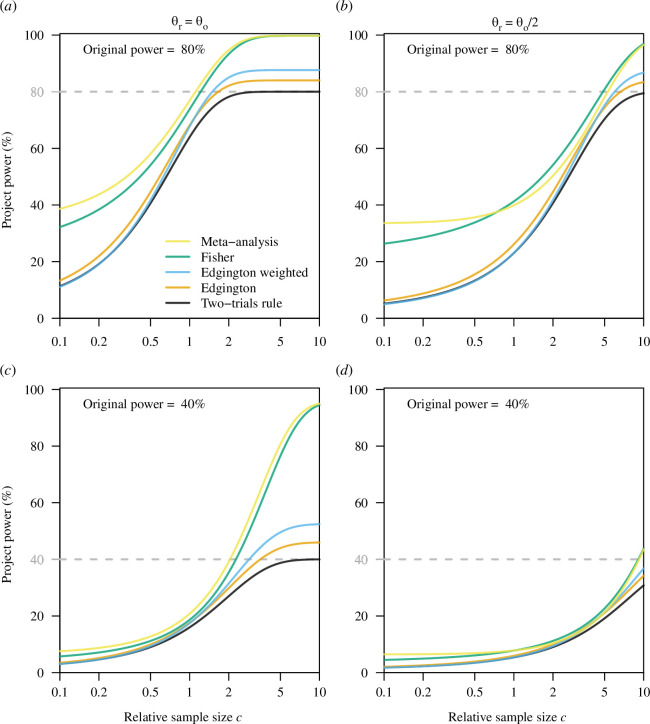
Project power of the two-trials rule, Edgington’s (both unweighted and weighted) and Fisher’s methods, and the meta-analysis criterion as a function of the relative sample size $c=n_{r}/n_{o}$ , assuming that the true effect sizes $\theta_{o}$ and $\theta_{r}$ are the same (*a,c*), or that the true replication effect size is half the true original effect size $\theta_{r}=\theta_{o}/2$ (*b,d*). The calculations assume that the original study has a power of 80% (*a*,*b*) or 40% (*c,d*) to detect the assumed true effect size at significance level α .

The project power is the probability to correctly declare replication success over both studies in combination when both underlying effects are non-null. Most original studies are designed to have 80% power to detect the assumed true effect size at significance level α , but the power can be considerably lower in reality [35,36]. In figure 1, we consider the project power with an original power of 80 % (figure 1*a*,*b*) and 40% (figure 1*c*,*d*), under the assumption that the true effect sizes are the same ( $\theta_{r}=\theta_{o}$ ; figure 1*a*,*c*) or that the true replication effect size is half the true original effect size ( $\theta_{r}=\theta_{o}/2$ ; figure 1*b*,*d*). The latter case reflects the shrinkage of effect estimates often encountered in replication projects.

In contrast to the conditional Type-I error rate, the project power of all methods depends on the original power to detect $\theta_{o}$ and the variance ratio c , which can often be interpreted as the relative sample size $c=n_{r}/n_{o}$ (replication to original); see appendix B for the derivations. The project power of the two-trials rule cannot become larger than the power in the original study to detect the true effect size (see figure 1). The project power of Edgington’s method is either essentially identical (for small c and the weighted version) or otherwise larger than the project power of the two-trials rule with limit 84% (unweighted) and 87.6% (weighted), respectively, for c→∞ and an original power of 80 %. The corresponding values are 46% (unweighted) and 52.5% (weighted) for an original power of 40 %. The project power of Fisher’s method and the meta-analysis criterion is larger than the project power of both the two-trials rule and Edgington’s method and reaches values close to 100% in the case $\theta_{r}=\theta_{o}$ even for a low original power of 40%. However, the price to pay is a considerable increase in conditional Type-I error rate, as discussed in §3.1.

## 4. Application

The *RPP* [5], the *EERP* [6], the *Social Sciences Replication Project* (*SSRP*) [7] and the *Experimental Philosophy Replicability Project* (*EPRP*) [8] are large-scale replication projects which aimed to replicate important discoveries published in journals from their respective fields. Here, we consider 138 original studies considered to have a ‘positive’ effect, i.e. either significant or with a ‘trend to significance’ (i.e. non-significant with a p -value just slightly larger than the significance threshold). Several methods were used to assess replication success, such as the two-trials rule, meta-analysis of the original and replication effect estimates or compatibility of the replication effect estimates with a prediction interval based on the original results.

**Figure 2.**
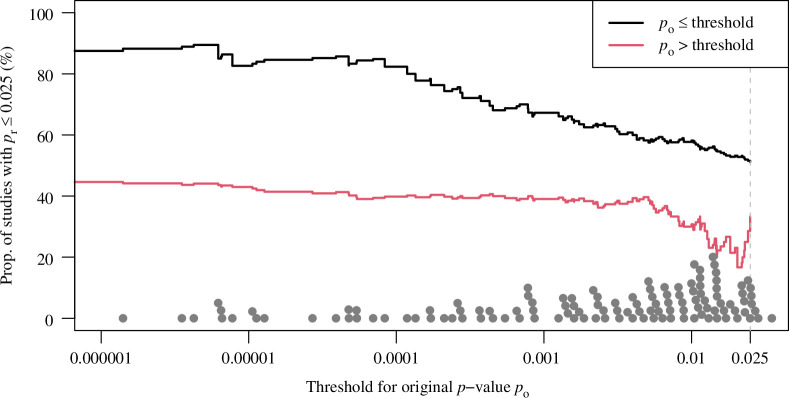
Proportion of significant replication studies ( $p_{r}\leq 0.025$ ) for original studies with p -value below and above the threshold, as a function of the threshold. The points at the bottom represent the original p -values $p_{o}$ . There are 17 study pairs with original p -value $p_{o}<0.000001$ that are not shown.

We grouped the 138 positive original studies into significant ( $p_{o}\leq$ threshold) and non-significant ( $p_{o}>$ threshold) at varying thresholds. The proportion of significant replication studies at the one-sided 0.025 level ( $p_{r}\leq \alpha =0.025$ ) is then calculated for each of the two groups and displayed in figure 2. The proportion of significant replication studies is higher for more convincing original studies (i.e. significant original studies at a smaller significance threshold). This shows that more convincing original studies tend to replicate more than less convincing original studies, and it therefore makes sense to be less stringent with the former as does Edgington’s method, but not the two-trials rule.

**Figure 3.**
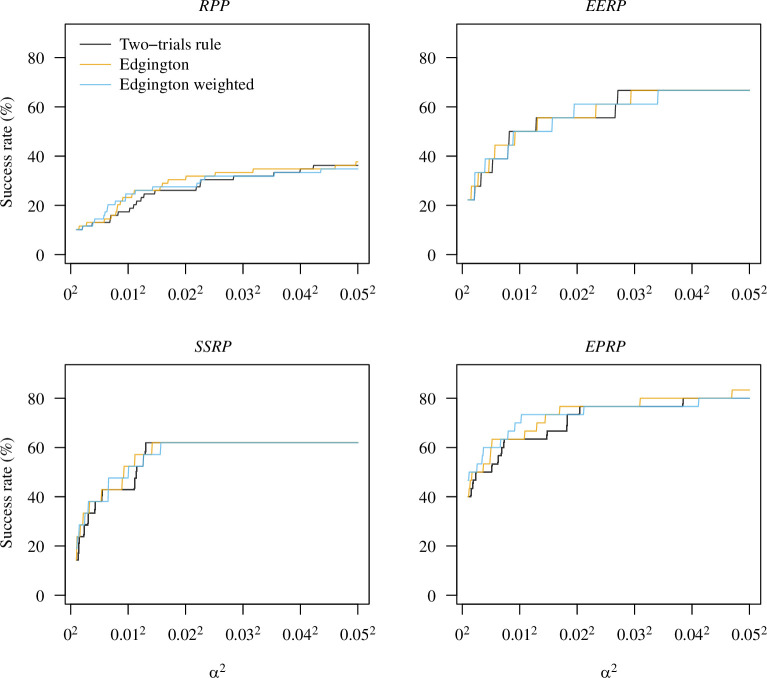
Success rate of the *RPP*, *EERP*, *SSRP* and *EPRP* as a function of the overall Type-I error rate $\alpha^{2}$ with the two-trials rule and Edgington’s method.

We then calculated the replication rates in the four projects with the two-trials rule ( $p_{\text{2TR}}\leq \alpha^{2}$ ) and Edgington’s method ( $p_{E}\leq \alpha^{2}$ or $p_{E_{w}}\leq \alpha^{2}$ ) for varying levels $\alpha^{2}$ (see figure 3). The replication rates are similar with a tendency of larger success rates with Edgington’s method. This is in line with the larger project power of Edgington’s method discussed in §3.2. For example, at level $\alpha^{2}=0.025^{2}$ , the replication rate of the two-trials rule and Edgington’s method are 30.4% versus 31.9% in the *RPP*, 55.6% versus 61.1% in the *EERP*, 61.9% for both in the *SSRP* and 76.7% for both in the *EPRP*. The conclusions only differ for two study pairs: the original study by Schmidt & Besner [37] and its replication in the *RPP*, and the original study by Ambrus & Greiner [13] and its replication in the *EERP*. As the original p -values $p_{o}=0.028$ and $p_{o}=0.027$ are slightly larger than α=0.025 in both cases, the two-trials rule is not fulfilled. However, as $p_{r}<0.0001$ and $p_{r}=0.006$ , respectively, the sum $p_{o}+p_{r}$ does not exceed b=0.035 and so replication success with Edgington’s method is achieved for both study pairs. Likewise, success is also achieved for the weighted method as $p_{o}+2\;p_{r}\leq 2\;\alpha =0.05$ in both cases.

**Figure 4.**
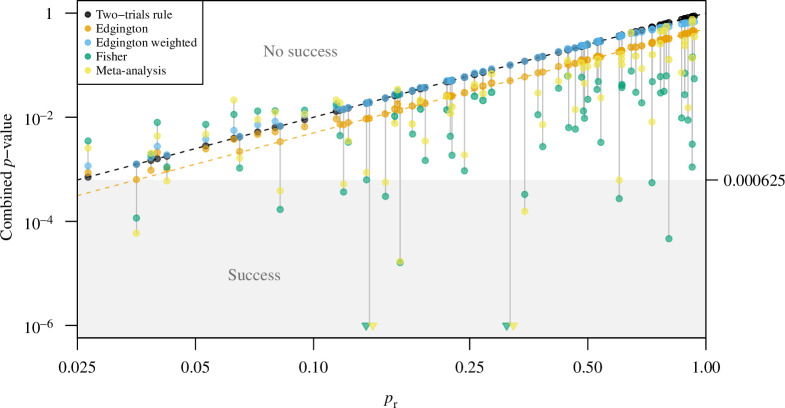
Combined p -values $p_{\text{2TR}}$ , $p_{E}$ , $p_{E_{w}}$ , $p_{F}$ and $p_{\text{MA}}$ have been calculated from the original and replication p -values $p_{o}$ and $p_{r}$ , respectively, for all replication studies considered in the four replication projects. They are plotted against the replication p -value $p_{r}$ for non-significant replication studies ( $p_{r}>0.025$ ). Combined p -values in the grey area flag replication success at overall Type-I error rate $0.025^{2}=0.000625$ . The dashed black line is the lower bound $p_{r}^{2}$ for $p_{\text{2TR}}$ . The dashed orange line is the lower bound $p_{r}^{2}/2$ for $p_{E}\leq 1/2$ . Fisher’s ( $p_{F}$ ) and meta-analytic ( $p_{\text{MA}}$ ) p -values smaller than 10^−6^ are marked with a triangle.

We also computed the combined p -values $p_{\text{2TR}}$ , $p_{E}$ , $p_{F}$ and $p_{\text{MA}}$ with each of the four methods and plotted them against the replication p -value $p_{r}$ for non-significant replication studies (see figure 4). By construction, the combined p -value from weighted Edgington’s method is always larger than the success threshold $\alpha^{2}=0.000625$ , and this is also the case for the unweighted version, where the smallest combined p -value $p_{E}=0.000635$ is just slightly above the threshold. In contrast, with Fisher’s method and the meta-analysis criterion, replication success is often declared although the replication p -value is quite large. There are even three studies with $p_{r}>0.5$ (so with an effect estimate in the wrong direction) which achieve success at level $\alpha^{2}=0.000625$ with Fisher’s method, and one study with the meta-analysis criterion. This illustrates that Fisher’s method and the meta-analysis criterion are not suited as a replacement for the two-trials rule.

## 5. Sample size calculation

The sample size of the replication study is usually calculated based on conditional power, i.e. such that the power 1−β to reach a significant replication effect estimate reaches a certain value, usually 80% or 90%, assuming the original effect estimate is the true one. If, additionally, significance of the original study is required, this corresponds to the sample size calculation based on the two-trials rule. In practice, a standard sample size calculation method is used where the minimal clinically important difference is replaced with the original effect estimate ${\hat{\theta }}_{o}$ . For example, for a balanced two-sample z -test, the sample size per group in the replication study is calculated as


(5.1) $$n_{r}=\frac{2\tau^{2}(z_{1-\alpha }+z_{1-\beta }{)}^{2}}{{\hat{\theta }}_{o}^{2}}\;,$$

where τ denotes the common standard deviation of the measurements and $z_{1-u}=\Phi^{-1}(1-u)$ denotes the 1−u quantile of the standard normal distribution. We note that in some replication projects [7] the original effect estimate ${\hat{\theta }}_{o}$ in (5.1) is reduced by 25% or even 50% to take into account its possible inflation [38].

It is also possible to express equation (5.1) on the relative scale. In that case, the required relative sample size $c=\sigma_{o}^{2}/\sigma_{r}^{2}=n_{r}/n_{o}$ is calculated as


(5.2) $$c=\frac{{\left(z_{1-\alpha }+z_{1-\beta }\right)}^{2}}{z_{o}^{2}}\;.$$

Sample size calculation based on (5.1) or (5.2) is appropriate if significance of the replication study at level α is the desired criterion for replication success. If instead Edgington’s method will be used, the sample size calculation needs to be appropriately adapted to ensure that the design of the replication study matches the analysis [39]. To do so, the significance level α needs to be replaced with $b-p_{o}$ in equations (5.1) and (5.2), so now depends on the p -value from the original study. A smaller sample size than with the two-trials rule is therefore required if $b-p_{o}>\alpha$ , i.e.


(5.3) $$p_{o}<b-\alpha =\sqrt{2}\;\alpha -\alpha =\alpha (\sqrt{2}-1)\approx 0.01\;.$$

The weighted version always requires a larger sample size than the two-trials rule, because the required significance level is $\alpha -p_{o}/2<\alpha$ .

**Figure 5.**
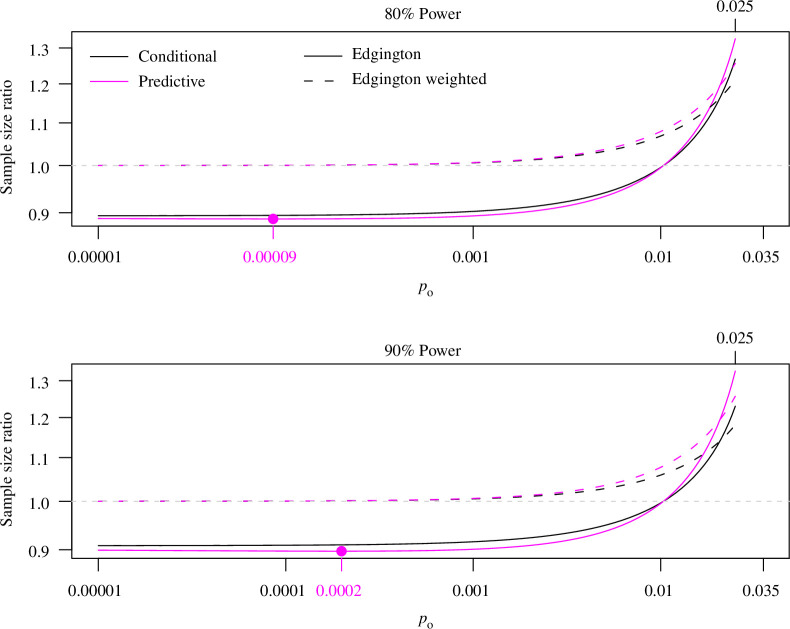
Replication sample size ratio of Edgington’s method compared with the two-trials rule to reach 80% (top) and 90% (bottom) power. For conditional power, the sample size ratio is monotonically increasing, while it reaches a minimum at $p_{o}=0.00009$ for predictive power in the unweighted version. The corresponding sample size reduction is one minus the sample size ratio. The sample size ratio of the weighted version is always monotonically increasing and converges to 1 for $p_{o}\to 0$ .


Figure 5 shows the sample size ratio

$$\frac{(z_{1-b+p_{o}}+z_{1-\beta }{)}^{2}}{(z_{1-\alpha }+z_{1-\beta }{)}^{2}}\;\text{resp.}\;\frac{(z_{1-\alpha +p_{o}/2}+z_{1-\beta }{)}^{2}}{(z_{1-\alpha }+z_{1-\beta }{)}^{2}}$$

of Edgington’s method (unweighted and weighted) versus the two-trials rule for α=0.025 , a power of 80% and 90% and $p_{o}\in [0.00001,0.025]$ . At 80% power, the sample size calculated with unweighted Edgington’s method can be up to 10.6% smaller than with the two-trials rule. At 90% power, the sample size reduction looks very similar with a maximum of 9.2%. However, if $p_{o}>0.01$ , the required sample size with Edgington’s method is larger than with the two-trials rule. The weighted version always requires a larger sample size, but smaller than the unweighted version if $p_{o}$ is close to α=0.025 .

A drawback of conditional power is that it does not take the uncertainty of the original result into account and hence can lead to underpowered replication studies. One way to take into account the uncertainty of the original result is to use ‘predictive power’ instead [40]. The relative sample size based on predictive power is generally larger than based on conditional power. The sample size reduction of Edgington’s method compared with the two-trials rule can be even more pronounced and reaches a value of 11.2% (10.3%) at $p_{o}=0.00009$ ( $p_{o}=0.0002$ ) for 80% (90%) predictive rather than conditional power (see figure 5).

## 6. Extensions to more than one replication study

It has been argued that a single replication study will often not be sufficient and that more than one replication study is needed to provide an unambiguous evaluation of replicability [41]. Edgington’s method can also be used if more than one replication study is conducted. A simple approach would be to combine the replication p -values into an overall replication p -value and then use Edgington’s method for one original and one replication p -value, as introduced in this article. However, Edgington’s method can also be applied directly to the individual p -values, as we now illustrate in the case of three studies (one original and two replications). Now the sum $E_{3}=p_{o}+p_{r1}+p_{r2}$ of the three p -values needs to be smaller than the new budget $b_{3}=0.16$ to ensure overall Type-I error control at level $0.025^{2}$ [32]. An interesting aspect of this approach is that it can be used to save resources if the replication studies are conducted sequentially. Indeed, there will be no point in conducting the second replication study if the sum of p -values $E_{2}=p_{o}+p_{r1}$ from the original and the first replication study is already larger than $b_{3}$ . Otherwise, a second replication study at significance level $b_{3}-E_{2}$ can be planned and we would flag replication success if $E_{3}=E_{2}+p_{r2}\leq b_{3}$ holds. Such a sequential conduct of replication studies has been suggested by Hedges & Schauer [41, p. 567] because ‘a single initial replication may be one effort in a sequence of replications, and as researchers conduct additional subsequent replications, eventually a preponderance of evidence will support more sensitive analyses’.

A refined version of this approach has been proposed by Held [32, §4], allowing to stop for success already after the first replication study. The approach is based on so-called alpha-spending [42], distributing the overall Type-I error rate $\alpha^{2}$ to the analysis after the first and the second replication study. Alpha-spending is a method originally proposed for interim analyses in clinical trials. Specifically, Fisher’s method has been proposed for the evaluation of experiments with an adaptive interim analysis based on the p -values of two subsamples of the study [43]. Closer to our approach is the method by Chang [44] who derives stopping rules based on the sum of the p -values for each subsample of the trial.

**Figure 6.**
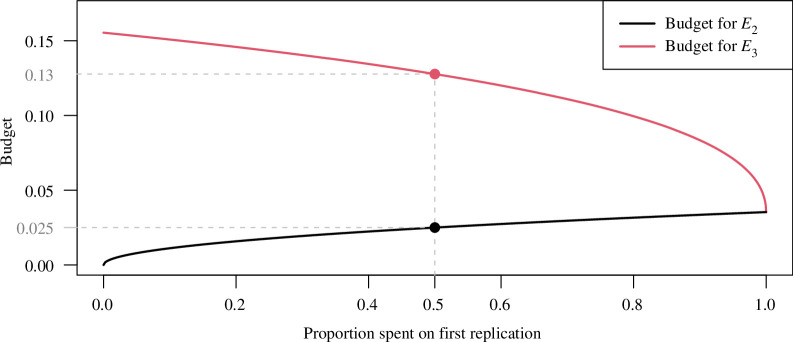
Budget $b_{2}$ for $E_{2}=p_{o}+p_{r1}$ and $b_{3}$ for $E_{3}=p_{o}+p_{r1}+p_{r2}$ , depending on the proportion of $\alpha^{2}=0.025^{2}$ spent on the first replication study. The points denote the available budget if half of $\alpha^{2}$ is spent after the first replication.

The application of the alpha-spending approach to the replication setting is illustrated in figure 6, which shows the budget $b_{2}$ for $E_{2}$ and $b_{3}$ for $E_{3}$ depending on the proportion of $\alpha^{2}$ that is spent on the analysis after the first replication. For example, if we spend half of $\alpha^{2}$ on the first replication, we can stop for replication success if $E_{2}=p_{o}+p_{r1}<b_{2}=0.025$ holds. If this is not the case but at least $E_{2}<b_{3}=0.13$ holds, we would plan and conduct a second replication study at significance level $b_{3}-E_{2}$ . If eventually $E_{3}=E_{2}+p_{r2}\leq b_{3}=0.13$ holds, we have achieved success after the second replication study. The combined procedure thus not only allows to stop for replication success or failure after the first replication study but offers a third possibility to conduct a second replication study if $b_{2}=0.025<E_{2}<b_{3}=0.13$ . The approach could also be extended to more than two replication studies and weights could also be introduced.

## 7. Discussion

We propose to use the sum of the p -values, also known as Edgington’s method, instead of the two-trials rule in the assessment of replication success. An unweighted and a weighted version are considered. In cases where it can be safely assumed that the original study follows the same standards as the replication study [45], we recommend to use the unweighted version. In cases where the original study may be subject to questionable research practices or publication bias, we recommend to give more weight to the replication study. The exact choice of the weight depends on how much we distrust the original study result. Both the unweighted and the weighted methods exactly control the overall Type-I error rate at level $\alpha^{2}$ and have an acceptable bound on the conditional Type-I error rate, namely, b=0.035 and α=0.025 , respectively. These numbers are for the conventional (but arbitrary) α=0.025 and a weight ratio of 2, and in principle, other values could be used.

The success bound for the replication p -value $p_{r}$ with Edgington’s method is not fixed at α=0.025 but varies between 0 and b=0.035 (unweighted) or between 0 and α=0.025 (weighted), depending on the original p -value $p_{o}$ . Replication success is possible for original studies that missed traditional statistical significance, as long as $p_{o}\leq 0.035$ (unweighted) or $p_{o}\leq 2\;\alpha =0.05$ (weighted). While these bounds are less stringent than with the two-trials rule, they are also different from the more elaborate sceptical p -value [46]. The sceptical p -value has been developed specifically for replication studies and depends on the two p -values, $p_{o}$ and $p_{r}$ , but also on the variance ratio, so treats original and replication studies not as exchangeable. The controlled sceptical p -value [47] ensures exact overall Type-I error rate control, just as all methods discussed in this article. It also allows for replication success if the original study is non-significant and can be used for sample size calculations. However, the method is more complicated and therefore more difficult to communicate. Edgington’s method can be seen as a simple compromise between the two-trials rule and the controlled sceptical p -value, valuing the combined evidence from both studies while ensuring that both studies support the alternative hypothesis. Of course, researchers may still want to quantify other aspects of replicability, such as statistical consistency of original and replication effect estimates, for which other methods, such as the Q -test, could be used [41]. In future work, we plan to conduct a simulation study to compare the operating characteristics of Edgington’s method with the sceptical p -value and alternative methods in the presence of publication bias and other questionable research practices [30,48].

One advantage of Edgington’s method is that it can be easily applied to non-normal or non-standard settings, for example, based on the t -test, a comparison of proportions or the log-rank test for survival data. For example, for a sample size calculation based on the t -test, the R function power.t.test() can be used to calculate the required replication sample size $n_{r}$ . The argument delta needs to be set to the original effect estimate ${\hat{\theta }}_{o}$ (perhaps incorporating some additional shrinkage) and the argument sig.level to $\sqrt{2}\;\alpha -p_{o}$ (Edgington) or $\alpha -p_{o}/2$ (weighted Edgington) rather than α (two-trials rule).

## Data Availability

The R package Replication Success available on CRAN at: https://CRAN.R-project.org/package=ReplicationSuccess has been used for the sample size calculations. The data of the *RPP*, *EERP*, *SSRP* and *EPRP* are available through the command data("RProjects"). All *p*-values have been recalculated based on Fisher’s *z*-transformation as described in [49], electronic supplementary material]; see also help("RProjects"). The code to reproduce the calculations in this paper is available at [50]. Functions to compute Edgington’s combined *p*-value (pEdgington) and associated power (powerEdgington) and sample size calculations (sampleSizeEdgington) are available in the development version of the ReplicationSuccess package which can be installed with remotes::install_github(repo = "crsuzh/ReplicationSuccess", ref = "edgington") (requires the remotes package available on CRAN).

## Appendix A. Weighted sum of *p*-values

The weighted sum of p -values (2.3) with weights $w_{o}\leq w_{r}$ can be written as

$$E_{w}=q_{o}+q_{r},$$

where $q_{o}∼U(0,w_{o})$ and $q_{r}∼U(0,w_{r})$ are independent uniform under the intersection null hypothesis. The density function of $E_{w}$ can be computed as the convolution of the densities of $q_{o}$ and $q_{r}$ and follows a trapezoidal distribution [51]:

$$f(e_{w})=\left\{ \begin{array}{rl} \frac{e_{w}}{w_{o}w_{r}} & \text{ if }0<e_{w}\leq w_{o}\;,\\ \frac{1}{w_{r}} & \text{ if }w_{o}<e_{w}\leq w_{r}\;,\\ \frac{w_{o}+w_{r}-e_{w}}{w_{o}w_{r}} & \text{ if }w_{r}<e_{w}\leq w_{o}+w_{r}\;. \end{array}\right.$$

The cumulative distribution function of $E_{w}$ is therefore

$$F(e_{w})=\left\{ \begin{array}{rl} \frac{e_{w}^{2}}{2\;w_{o}w_{r}} & \text{ if }0<e_{w}\leq w_{o}\;,\\ \frac{1}{w_{r}}\left(e_{w}-\frac{w_{o}}{2}\right) & \text{ if }w_{o}<e_{w}\leq w_{r}\;,\\ 1+\frac{1}{w_{o}w_{r}}\left(e_{w}(w_{o}+w_{r})-\frac{(w_{o}+w_{r}{)}^{2}}{2}-\frac{e_{w}^{2}}{2}\right) & \text{ if }w_{r}<e_{w}\leq w_{o}+w_{r}\;. \end{array}\right.$$

A valid combined p -value is obtained by plugging $E_{w}$ into the cumulative distribution function: $p_{E_{w}}=F(E_{w})$ .

## Appendix B. Project power

First assume that both effects are the same, so $\theta_{r}=\theta_{o}$ . As a result, $z_{o}∼N(\mu ,1)$ and $z_{r}∼N(\mu \sqrt{c},1)$ . The project power of the two-trials rule can be found in Held *et al.* [52, §3.3]. The project power of all other methods is calculated as

$$\int \mathrm{Pr}(\text{Replication success}|z_{o})\phi (z_{o}-\mu )\;dz_{o}.$$

Specifically, Edgington’s method has project power

(B 1)
$$\int_{\Phi^{-1}(1-b)}^{\infty }\;\mathrm{Pr}(p_{r}\leq b-p_{o})\phi (z_{o}-\mu )\;dz_{o}\;\;\int_{\Phi^{-1}(1-b)}^{\infty }\;\mathrm{Pr}(z_{r}\geq \Phi^{-1}(1-b+p_{o}))\phi (z_{o}-\mu )\;dz_{o}\;\;\int_{\Phi^{-1}(1-b)}^{\infty }\;\mathrm{Pr}(z_{r}\geq \Phi^{-1}(2-b-\Phi (z_{o})))\phi (z_{o}-\mu )\;dz_{o}\;\;=\int_{\Phi^{-1}(1-b)}^{\infty }\;\left[1-\Phi \left\{\Phi^{-1}\left(2-b-\Phi (z_{o})\right)-\mu \sqrt{c}\right\}\right]\phi (z_{o}-\mu )\;dz_{o}.$$

The project power of weighted Edgington’s method with weight ratio $\tilde{w}=w_{r}/w_{o}$ turns out to be

(B 2)
$$\int_{\Phi^{-1}(1-b_{w}/w_{o})}^{\infty }\left[1-\Phi \left\{\Phi^{-1}\left(1+1/\tilde{w}-\sqrt{2/\tilde{w}}\;\alpha -\Phi (z_{o})/\tilde{w}\right)-\mu \sqrt{c}\right\}\right]\phi (z_{o}-\mu )\;dz_{o}\;.$$

The project power of Fisher’s method is

(B 3)
$$\begin{array}{l} \int_{0}^{\infty }f(z_{o})\;\phi (z_{o}-\mu )\;dz_{o}\text{ with }f(z_{o})=\left\{ \begin{array}{ll} 1-\Phi \left\{\Phi^{-1}\left(1-\frac{c_{F}}{1-\Phi (z_{o})}\right)-\mu \sqrt{c}\right\}, & \text{if }1-\Phi (z_{o})\geq c_{F}\\ 1, & \text{otherwise}\;, \end{array}\right. \end{array}$$

and the project power of the meta-analysis criterion is

(B 4)
$$\int_{0}^{\infty }\left[1-\Phi \;\;\left\{\frac{\Phi^{-1}(1-\alpha^{2})\sqrt{c+1}-z_{o}}{\sqrt{c}}\right\}-\mu \sqrt{c}\right]\phi (z_{o}-\mu )\;dz_{o}\;.$$

The project power of Edington’s method converges to

$$1-\Phi \;\;\left(\Phi^{-1}(1-b)-\Phi^{-1}(1-\alpha )-\Phi^{-1}(1-\beta )\right)$$

for c→∞ , while the corresponding limit is

$$1-\Phi \;\;\left(\Phi^{-1}(1-b_{w}/w_{o})-\Phi^{-1}(1-\alpha )-\Phi^{-1}(1-\beta )\right)$$

for weighted Edgington’s method and 1 for the other two methods.

If $\theta_{r}=\theta_{o}/2$ , the term $\mu \sqrt{c}$ in (B 1), (B 2), (B 3) and (B 4) needs to be divided by 2 .
